# Supplementary material for: Risk of Anxiety and Depression in Patients with Inflammatory Bowel Disease: A Nationwide, Population-Based Study
Source: J Clin Med. 2019 May 10;8(5):654. doi: 10.3390/jcm8050654 (PMC6572298; doi:10.3390/jcm8050654)
Supplement: Supplementary file 1 [file jcm-08-00654-s001.pdf]

Supplementary Table S1. Incidence and risk of anxiety based on medication use among patients with inflammatory bowel disease (IBD).

	N	Events (n)	Follow-up duration (person-years)	Incidence rate (per 1,000 person-years)	Adjusted HR* (95% CI)
CD					
Immunomodulators					
No	2,531	285	11,754.87	24.25	1 (Ref)
Yes	3,865	332	17,835.02	18.62	0.87 (0.74-1.02)
Steroids					
No	2,707	206	13,352.60	15.43	1 (Ref)
Yes	3,689	411	16,237.29	25.31	1.71 (1.45-2.03)
Biologics					
No	5,464	532	25,330.33	21.00	1 (Ref)
Yes	932	85	4,259.56	19.96	1.01 (0.80-1.28)
UC					
Immunomodulators					
No	7,844	1,110	35,708.14	31.09	1 (Ref)
Yes	1,329	177	6,056.7	29.22	1.03 (0.88-1.21)
Steroids					
No	4,181	494	19,771.87	24.99	1 (Ref)
Yes	4,992	793	21,992.97	36.06	1.44 (1.28-1.61)
Biologics					
No	8,983	1,262	41,048.41	30.74	1 (Ref)
Yes	190	25	716.42	34.90	1.28 (0.86-1.91)

CD, Crohn's disease; CI, Confidence interval; HR, Hazard ratio; Ref, reference; UC, Ulcerative colitis.

*Adjusted by age, sex, residence, income, and comorbid medical conditions including diabetes mellitus, hypertension, dyslipidemia, congestive heart failure, ischemic heart disease, chronic pulmonary obstructive disease, cerebrovascular disease, end-stage renal disease, and malignancy

Supplementary Table S2. Incidence and risk of depression based on medication use among patients with IBD.

	N	Events (n)	Follow-up duration (person-years)	Incidence rate (per 1,000 person-years)	Adjusted HR* (95% CI)
CD					
Immunomodulators					
No	2,531	191	12,022.67	15.89	1 (Ref)
Yes	3,865	246	18,025.99	13.65	0.96 (0.79-1.17)
Steroids					
No	2,707	150	13,512.94	11.10	1 (Ref)
Yes	3,689	287	16,535.72	17.36	1.58 (1.29-1.93)
Biologics					
No	5,464	353	25,804.89	13.68	1 (Ref)
Yes	932	84	4,243.77	19.79	1.51 (1.18-1.93)
UC					
Immunomodulators					
No	7,844	659	37,116.31	17.76	1 (Ref)
Yes	1,329	148	6,159.78	24.03	1.49 (1.24-1.78)
Steroids					
No	4,181	330	20,255.90	16.29	1 (Ref)
Yes	4,992	477	23,020.20	20.72	1.26 (1.10-1.45)
Biologics					
No	8,983	781	42,545.69	18.36	1 (Ref)
Yes	190	26	730.40	35.60	2.23 (1.50-3.30)

CD, Crohn's disease; CI, Confidence interval; HR, Hazard ratio; Ref, reference; UC, Ulcerative colitis.

*Adjusted by age, sex, residence, income, and comorbid medical conditions including diabetes mellitus, hypertension, dyslipidemia, congestive heart failure, ischemic heart disease, chronic pulmonary obstructive disease, cerebrovascular disease, end-stage renal disease, and malignancy
